# Melatonin Pre-Treatment Protects Erythrocytes Against Subsequent Oxidative Damage

**DOI:** 10.3390/molecules30030658

**Published:** 2025-02-01

**Authors:** Tomas Jasenovec, Rastislav Vazan, Dominika Radosinska, Roman Gardlik, Jana Radosinska

**Affiliations:** 1 Institute of Physiology, Faculty of Medicine, Comenius University in Bratislava, Sasinkova 2, 811 08 Bratislava, Slovakia; tomas.jasenovec@fmed.uniba.sk (T.J.); rastislav.vazan@fmed.uniba.sk (R.V.); dominika.radosinska@fmed.uniba.sk (D.R.); 2 Institute of Medical Biology, Genetics and Clinical Genetics, Faculty of Medicine, Comenius University in Bratislava, Sasinkova 4, 811 08 Bratislava, Slovakia; 3 Institute of Molecular Biomedicine, Faculty of Medicine, Comenius University in Bratislava, Sasinkova 4, 811 08 Bratislava, Slovakia; roman.gardlik@imbm.sk

**Keywords:** erythrocytes, melatonin, tert-butyl hydroperoxide, erythrocyte deformability, osmotic resistance, oxidative stress

## Abstract

Research on the effects of melatonin on erythrocyte deformability has yielded mixed results. While some studies reported improvements, others found no effect, and a few even noted a deterioration in deformability. Moreover, the impact of melatonin may vary between healthy erythrocytes and those subjected to oxidative stress. This study investigated the dose-dependent effects of melatonin on erythrocytes under baseline conditions and oxidative stress, using both pre- and post-stress incubation protocols. Oxidative damage was induced with tert-butyl hydroperoxide (TBHP), and its extent was assessed via dichlorofluorescein fluorescence. Erythrocyte deformability was measured using ektacytometry, and osmotic resistance was assessed through hemolytic assays. The results showed that incubation with TBHP led to a dose-dependent decline in both erythrocyte deformability and osmotic resistance. While melatonin treatment had no observable effect on intact erythrocytes, it enhanced deformability in oxidatively damaged erythrocytes when administered before oxidative stress was induced. However, the beneficial effect was not evident when melatonin was applied after oxidative damage. Additionally, melatonin incubation had no impact on the ability of erythrocytes to resist the hypotonic environment. In conclusion, this study supports the notion that the antioxidant properties of melatonin can improve erythrocyte functional status, as reflected by enhanced deformability, but not osmotic resistance. Notably, this effect was observed only in erythrocytes that were exposed to oxidative damage after melatonin incubation, not in intact cells.

## 1. Introduction

Melatonin is best known as a hormone produced by the pineal gland and as a substance with pleiotropic effects. In addition, it is widely produced in nature and is present in many nutritional products [1]. While its primary role is the regulation of biological rhythms, it also exhibits potent antioxidant properties. Melatonin acts as a direct scavenger of free radicals, reducing oxidative stress [2]. Additionally, it stimulates the activity or expression of endogenous antioxidant cellular enzymes, including superoxide dismutase, catalase, and glutathione peroxidase in various tissues (e.g., brain, liver, and kidney), which protects them against oxidative damage [3].

Melatonin is secreted in response to darkness, leading to typical circadian variations in its concentration in blood plasma. The highest levels occur at night, usually between 2:00 and 4:00 a.m. Both the peak values and the intensity of these oscillations vary depending on age. Infants under three months have negligible melatonin concentration in blood plasma, which increases and becomes circadian in older infants. Peak nocturnal levels are highest at 1–3 years (average 1400 pM, i.e., 325 pg/mL) and thereafter decline. In young adults, daytime and nighttime averages are 40 and 260 pM (10 and 60 pg/mL), respectively [4]. Oral supplementation of melatonin significantly increases its levels in the bloodstream. For example, a 3 mg dose of melatonin raised serum levels to more than 100 times the baseline concentration within 20 min, increasing from 21.6 pg/mL at baseline to 3561 pg/mL. This marked increase persisted 60 min following ingestion, while the level was approximately 131 pg/mL after 4 h [5].

In studies investigating the effects of various molecules with potential antioxidant properties, erythrocytes are commonly used [6]. Their primary function is the transport of oxygen, enabled by their high hemoglobin concentration. The presence of iron ions in reduced (Fe^2+^) form within a molecule of hemoglobin, combined with the proximity to oxygen, subjects erythrocytes to a constant risk of oxidative damage. To counteract this, erythrocytes possess sophisticated antioxidant defense mechanisms. They can be divided into three categories: (1) antioxidant molecules and redox pairs, such as ascorbate and glutathione in its reduced (GSH) and oxidized (GSSG) forms, (2) redox equivalents—NADH and NADPH, and (3) antioxidant enzymes [7]. When erythrocytes are unable to effectively manage oxidative damage, their functional status becomes compromised [8]. This is a characteristic feature of pathophysiological conditions in which erythrocytes may even act as mediators of disease processes [9,10]. Oxidative damage to erythrocytes disrupts cytoskeletal proteins, resulting in increased membrane stiffness and, ultimately, hemolysis [11]. Thus, redox metabolism represents a key aspect of overall erythrocyte metabolism [12].

Due to erythrocyte sensitivity to various stimuli and the ready availability of blood samples, erythrocytes have been proposed as valuable cellular models for assessing the cytoprotective or cytotoxic effects of tested substances [6,13]. Although erythrocytes are not only beneficial as a model for studying the biological effects of various molecules, they also merit protection against oxidative damage to preserve their role as physiological regulators. Improving erythrocyte quality also remains an ongoing challenge in optimizing blood storage conditions. A commonly used method to assess the functional status of erythrocytes is by evaluating their deformability—their ability to change shape and pass through different parts of the circulatory system under varying hemodynamic conditions. Research on the effects of melatonin on erythrocyte deformability has produced mixed results, with some studies reporting improvements [14,15], others finding no effect [16], and a few even noting deterioration in deformability [17,18].

The effects of treatment, including melatonin intervention, may differ between healthy erythrocytes and those subjected to oxidative impairment. In in vitro experiments, various substances are used to induce oxidative damage to erythrocytes, including hydrogen peroxide [19,20], tert-butyl hydroperoxide (TBHP) [6,21,22], cumene hydroperoxide [23], or bisphenol A [24]. Each of these agents has specific advantages and limitations. In this study, the relatively well-characterized effects of TBHP were leveraged as a methodological advantage. TBHP is an organic hydroperoxide that is a water-soluble peroxide capable of diffusing through cell membranes [25], making it widely used in oxidation reaction studies. Upon exposure to TBHP, erythrocytes undergo lipid peroxidation and hemoglobin oxidation, processes significantly influenced by GSH intracellular stores [26]. Once GSH is depleted, TBHP induces characteristic disruption in erythrocyte cytoskeletal proteins, including spectrin, actin, and protein 4.1 [25]. These changes can lead to an increased extent of hemolysis, as has been observed in erythrocytes from younger individuals [27].

**Figure 1:**
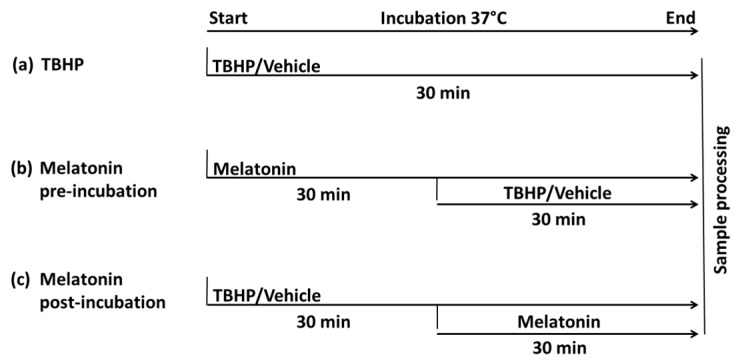
Design of in vitro experiments. (**a**) The effect of different TBHP concentrations; (**b**) the effect of melatonin pre-incubation followed by oxidative damage; (**c**) the effect of melatonin on erythrocytes already subjected to oxidative damage. Abbreviation: TBHP—tert-butyl hydroperoxide.

Building on the well-documented effects of TBHP on erythrocytes and addressing the inconsistent findings regarding the impact of melatonin on erythrocytes, this in vitro study aimed to comprehensively investigate the effect of melatonin. Specifically, this study sought to assess the melatonin effect on erythrocytes under baseline conditions, as well as under oxidative stress, with pre- and post-stress incubation protocols (Figure 1).

To achieve these objectives, the study is structured into the following subtasks:

(a) Evaluating the extent of oxidative damage to erythrocytes and its impact on erythrocyte deformability and osmotic resistance across varying concentrations of TBHP;
(b) The main part—examining the effects of melatonin at varying concentrations on erythrocytes, as well as on the level of oxidative damage following their pre-incubation with melatonin;
(c) Assessing the effects of melatonin administered following TBHP-induced oxidative damage.

## 2. Results

### 2.1. The Evaluation of Oxidative Damage Intensity and Its Impact on Erythrocyte Quality at Varying Concentrations of TBHP

The initial phase of the study (Figure 1a) focuses on assessing oxidative damage to erythrocytes by measuring dichlorofluorescein (DCF) fluorescence and examining its impact on erythrocyte functionality through deformability and osmotic resistance tests. The data obtained in this set of experiments were evaluated using one-way ANOVA.

#### 2.1.1. The Impact of Different TBHP Concentrations on Free Radical Formation in Erythrocytes

**Figure 2:**
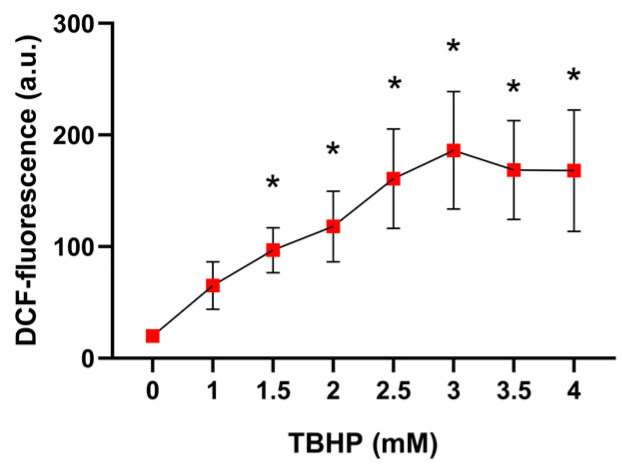
The effect of a 30 min exposure of erythrocytes to various concentrations of TBHP on DCF-related fluorescence in erythrocytes. Abbreviations: DCF—dichlorofluorescein, TBHP—tert-butyl hydroperoxide. Data are presented as means ± standard deviations. Statistical significance: * *p* < 0.05 compared with TBHP concentration of 0 mM.

The 30 min exposure of erythrocytes to TBHP resulted in a dose-dependent increase in DCF-related fluorescence in erythrocytes (*p* = 0.0018). This effect reached statistical significance at TBHP concentrations of 1.5 mM and higher. Beyond approximately 2.5 mM, the fluorescence signal plateaued (Figure 2).

#### 2.1.2. The Impact of Different TBHP Concentrations on Erythrocyte Deformability

**Figure 3:**
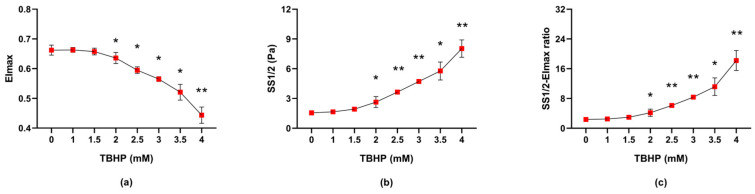
The effect of a 30 min exposure of erythrocytes to various concentrations of TBHP on erythrocyte deformability parameters: (**a**) EImax, (**b**) SS1/2 and (**c**) SS1/2-EImax ratio. Abbreviations: EImax—maximal elongation index, SS1/2—shear stress corresponding to half-maximal elongation, TBHP—tert-butyl hydroperoxide. Data are presented as means ± standard deviations. Statistical significance: * *p* < 0.05, ** *p* < 0.01 compared with TBHP concentration of 0 mM.

The maximal elongation index (EImax) decreased with increasing concentration of TBHP (*p* = 0.0002), with statistical significance observed at TBHP of 2 mM and above (Figure 3a). Similarly, the shear stress corresponding to half-maximal elongation (SS1/2) increased with TBHP concentration (*p* < 0.0001), also reaching statistical significance from 2 mM onward (Figure 3b). When the ratio of EImax and SS1/2 was assessed—an approach considered by Baskurt and Meiselman [28] to more robustly reflect changes in deformability across different erythrocyte populations—the results followed the same trend (*p* = 0.0002), with 2 mM being the first TBHP concentration to show a statistically significant difference compared with the absence of TBHP (Figure 3c).

#### 2.1.3. The Impact of Different TBHP Concentrations on Erythrocyte Osmotic Resistance

**Figure 4:**
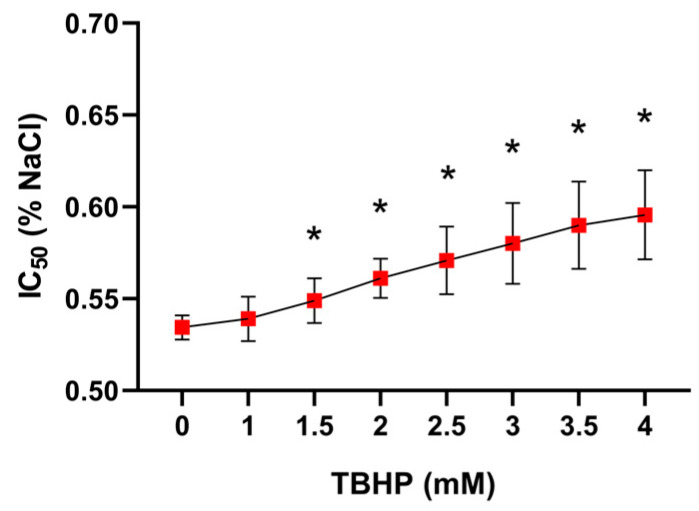
The effect of a 30 min exposure of erythrocytes to various concentrations of TBHP on osmotic resistance of erythrocytes. Abbreviations: IC_50_—NaCl concentration in which 50% hemolysis occurs, TBHP—tert-butyl hydroperoxide. Data are presented as means ± standard deviations. Statistical significance: * *p* < 0.05 compared with TBHP concentration of 0 mM.

The erythrocyte osmotic resistance is expressed in the form of an IC_50_ value that represents the NaCl concentration in which 50% hemolysis occurs. The higher IC_50_ value corresponds to the lower ability of erythrocytes to survive in a hypotonic solution. The IC_50_ value increased with increasing concentrations of TBHP (*p* = 0.0026), with the effect becoming apparent even at 1.5 mM (Figure 4).

### 2.2. The Effects of Melatonin Across a Range of Concentrations on Both Control and Oxidatively Damaged Erythrocytes

The same methodical approaches used in the first set of experiments (Section 2.1) were also applied in this part of the study. These measurements were performed following pre-incubation with melatonin and included both control erythrocytes (unaffected by oxidative stress) and oxidatively impaired erythrocytes incubated with 2.5 mM TBHP (Figure 1b). The data obtained in this set of experiments were evaluated using two-way ANOVA with repeated measures.

#### 2.2.1. The Effect of Melatonin Across a Range of Concentrations on Free Radical Formation in Erythrocytes Following Pre-Incubation with Melatonin and Subsequent Exposure to TBHP

**Figure 5:**
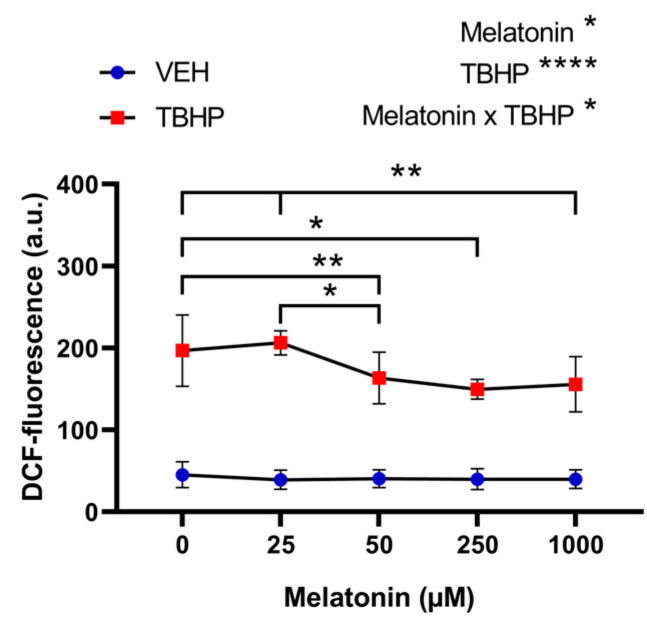
The effect of melatonin at various concentrations on DCF-related fluorescence in erythrocytes, following a 30 min pre-incubation with melatonin, and subsequent exposure to either vehicle or TBHP (2.5 mM) for an additional 30 min. Abbreviations: DCF—dichlorofluorescein, VEH—vehicle (TBHP absence), TBHP—tert-butyl hydroperoxide. Data are presented as means ± standard deviations. Statistical significance: * *p* < 0.05, ** *p* < 0.01; two-way ANOVA: * *p* < 0.05, **** *p* < 0.0001.

The two-way ANOVA with repeated measures revealed a significant effect of both factors: melatonin concentration (*p* = 0.0104) and TBHP (*p* < 0.0001), as well as their interaction (*p* = 0.0254), on the levels of free radicals detected by DCF fluorescence. No significant differences were observed among the groups of erythrocytes that were not exposed to TBHP. However, in oxidatively damaged erythrocytes, significantly fewer free radicals were detected with melatonin pre-treatment at concentrations of 50 μM (*p* = 0.0099), 250 μM (*p* = 0.0263), and 1000 μM (*p* = 0.0012) when compared with erythrocytes not exposed to melatonin. Additionally, erythrocytes treated with 250 μM (*p* = 0.0483) and 1000 μM (*p* = 0.0092) of melatonin exhibited significantly lower free radical levels compared with the group treated with 25 μM (Figure 5).

#### 2.2.2. The Effect of Melatonin Across a Range of Concentrations on Deformability of Erythrocytes Following Pre-Incubation with Melatonin and Subsequent Exposure to TBHP

**Figure 6:**
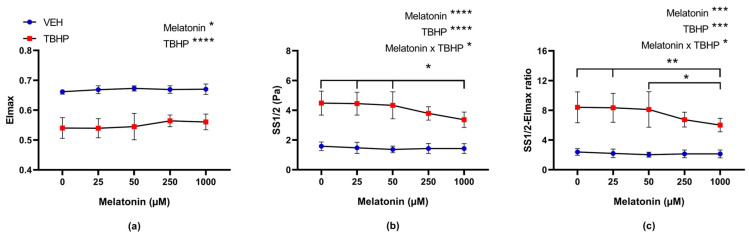
The effect of melatonin at various concentrations on erythrocyte deformability parameters: (**a**) EImax, (**b**) SS1/2 and (**c**) SS1/2-EImax ratio, following a 30 min pre-incubation with melatonin, and subsequent exposure to either vehicle or TBHP (2.5 mM) for an additional 30 min. Data are presented as means ± standard deviations. Abbreviations: EImax—maximal elongation index, SS1/2—shear stress corresponding to half-maximal elongation, VEH—vehicle (TBHP absence), TBHP—tert-butyl hydroperoxide. Statistical significance: * *p* < 0.05, ** *p* < 0.01; two-way ANOVA: * *p* < 0.05, *** *p* < 0.001, **** *p* < 0.0001.

When focusing on EImax, data analysis revealed a significant effect of melatonin concentration (*p* = 0.0013) and oxidative damage caused by TBHP (*p* < 0.0001). A lower EImax value in TBHP-incubated erythrocytes indicates a significant and expected reduction in their deformability following oxidative damage. Additionally, the multiple comparison tests revealed that melatonin had no significant effect on either oxidatively damaged erythrocytes or control erythrocytes (Figure 6a). The analysis also showed that the shear stress required for half-maximal EImax (SS1/2) was significantly influenced by both factors: melatonin concentration (*p* < 0.0001) and oxidative damage caused by TBHP (*p* < 0.0001), as well as their interaction (*p* = 0.0379). No differences were detected among control (TBHP absence) erythrocytes (Figure 6b). In oxidatively damaged groups, a significant decrease in SS1/2 was observed in the group treated with 1000 μM of melatonin compared to the group without melatonin exposure (*p* = 0.0038), as well as in groups treated with 25 μM (*p* = 0.0051) and 50 μM (*p* = 0.0137) of melatonin (Figure 6b).

When the ratio of SS1/2 to EImax was evaluated, melatonin concentration (*p* = 0.0004), TBHP exposure (*p* = 0.0002), and their interaction (*p* = 0.0304) were identified as statistically significant in a two-way ANOVA with repeated measures analysis. Differences among oxidatively damaged groups became more pronounced, particularly when comparisons were made with the group treated with 1000 μM of melatonin, as indicated by lower *p*-values (Figure 6c).

#### 2.2.3. The Effect of Melatonin Across a Range of Concentrations on the Osmotic Resistance of Erythrocytes Following Pre-Incubation with Melatonin and Subsequent Exposure to TBHP

**Figure 7:**
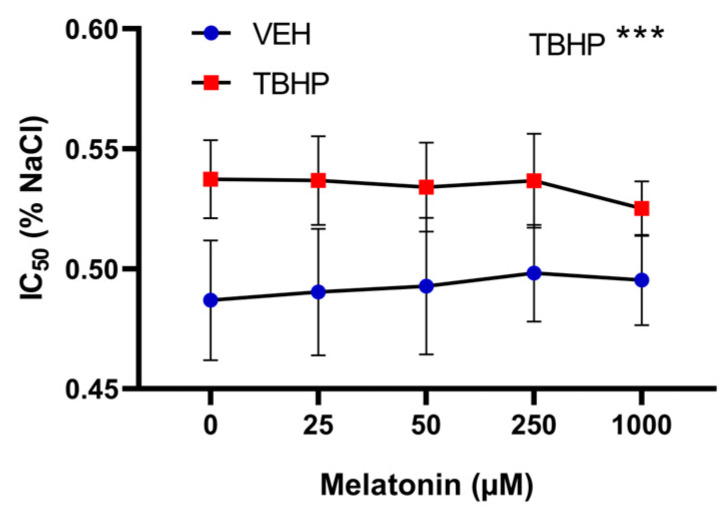
The effect of melatonin at various concentrations on osmotic resistance of erythrocytes following a 30 min pre-incubation with melatonin, and subsequent exposure to either vehicle or TBHP (2.5 mM) for an additional 30 min. Data are presented as means ± standard deviations. Abbreviations: IC_50_—NaCl concentration in which 50% hemolysis occurs, VEH—vehicle (TBHP absence), TBHP—tert-butyl hydroperoxide. Statistical significance: two-way ANOVA: *** *p* < 0.001.

Using two-way ANOVA with repeated measures, oxidative damage induced by TBHP was identified as a significant factor influencing erythrocyte osmotic resistance (*p* = 0.0003). However, melatonin pre-treatment did not significantly influence the IC_50_ parameter (*p* = 0.293). When evaluating differences within groups, no effect of melatonin on osmotic resistance was observed in either oxidatively undamaged erythrocytes or those damaged by TBHP (Figure 7).

### 2.3. The Effects of Melatonin Administered Following TBHP-Induced Oxidative Damage

In the final part of the study, measurements were conducted on both control erythrocytes (unaffected by oxidative stress) and oxidatively impaired erythrocytes incubated with 2.5 mM TBHP. In this phase, oxidative stress was induced 30 min before adding the melatonin solution to the incubation medium (Figure 1c). The data obtained in this set of experiments were evaluated using two-way ANOVA with repeated measures.

#### 2.3.1. The Effect of Melatonin on Free Radical Formation in Control and Oxidatively Impaired Erythrocytes

**Figure 8:**
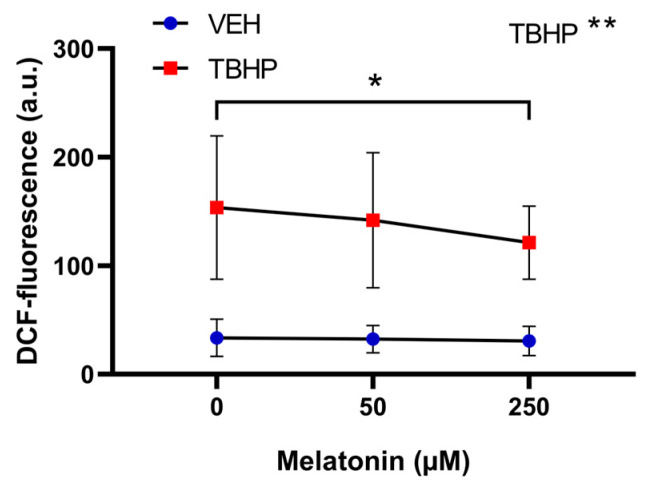
The effect of melatonin at various concentrations on DCF-related fluorescence in erythrocytes following a 30 min exposure to either vehicle or TBHP (2.5 mM), and subsequent post-incubation with melatonin for additional 30 min. Data are presented as means ± standard deviations. Abbreviations: DCF—dichlorofluorescein, VEH—vehicle (TBHP absence), TBHP—tert-butyl hydroperoxide. Statistical significance: * *p* < 0.05, two-way ANOVA: ** *p* < 0.01.

For erythrocyte DCF-related fluorescence, the two-way ANOVA with repeated measures revealed only TBHP as a significant factor (*p* = 0.0014). In multiple comparison tests, there was a statistically significant difference only when comparing the oxidatively damaged erythrocytes with the highest melatonin concentration (250 µM) to erythrocytes with no melatonin added (*p* = 0.0301) (Figure 8).

#### 2.3.2. The Effect of Melatonin on Deformability of Control and Oxidatively Impaired Erythrocytes

**Figure 9:**
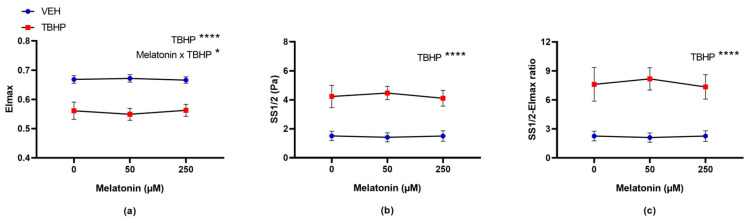
The effect of melatonin at various concentrations on erythrocyte deformability parameters: (**a**) EImax, (**b**) SS1/2 and (**c**) SS1/2-EImax ratio, following a 30 min exposure to either vehicle or TBHP (2.5 mM), and subsequent post-incubation with melatonin for additional 30 min. Data are presented as means ± standard deviations. Abbreviations: EImax—maximal elongation index, SS1/2—shear stress corresponding to half-maximal elongation, VEH—vehicle (TBHP absence), TBHP—tert-butyl hydroperoxide. Statistical significance: two-way ANOVA: * *p* < 0.05, **** *p* < 0.0001.

The two-way ANOVA with repeated measures identified a significant effect of TBHP (*p* < 0.0001) and the interaction between melatonin and TBHP (*p* = 0.0480) on the EImax value (Figure 9a). For the SS1/2 value and the ratio EImax to SS1/2 parameters, only TBHP-induced oxidative damage was identified as a significant factor (*p* < 0.0001 for both) (Figure 9b,c). Multiple comparison tests did not detect any significant differences in parameters across the various melatonin concentrations in either oxidatively damaged or control erythrocytes (Figure 9).

#### 2.3.3. The Effect of Melatonin on Osmotic Resistance of Control and Oxidatively Impaired Erythrocytes

**Figure 10:**
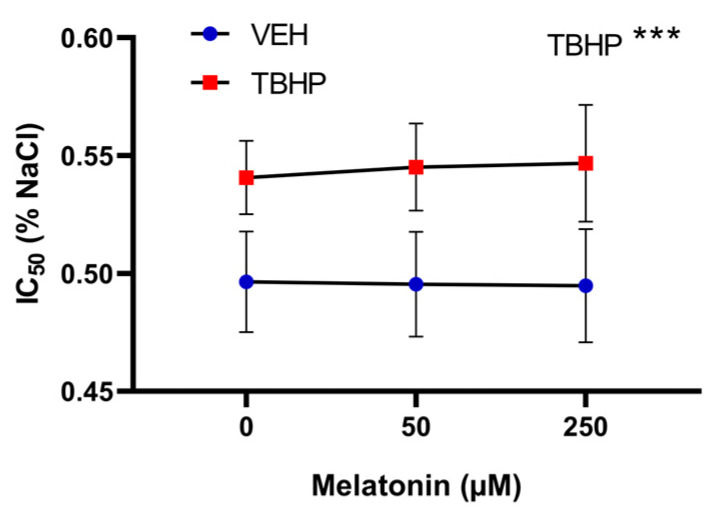
The effect of melatonin at various concentrations on osmotic resistance of erythrocytes following a 30 min exposure to either vehicle or TBHP (2.5 mM), and subsequent post-incubation with melatonin for additional 30 min. Data are presented as means ± standard deviations. Abbreviations: IC_50_—NaCl concentration in which 50% hemolysis occurs, VEH—vehicle (TBHP absence), TBHP—tert-butyl hydroperoxide. Statistical significance: two-way ANOVA: *** *p* < 0.001.

For osmotic resistance, oxidative damage induced by TBHP was the only factor identified as significant (*p* = 0.0001). No effect of melatonin on osmotic resistance was observed when analyzing group differences (Figure 10).

## 3. Discussion

The first part of this study aimed to assess the intensity of oxidative damage at varying concentrations of TBHP and demonstrates that incubation of erythrocytes with TBHP resulted in dose-dependent deterioration of erythrocyte deformability and osmotic resistance. The first statistically significant differences when compared with the control (i.e., incubation in the absence of TBHP) occurred at a TBHP concentration of around 2 mM. A more sensitive parameter was erythrocyte osmotic resistance, in which a TBHP concentration of 1.5 mM induced its deterioration. In further experimental parts, this study used a TBHP concentration of 2.5 mM. This concentration was not regarded as the first significant level but was chosen because it reliably induced oxidative damage. At this concentration, the erythrocyte status showed clear signs of damage, but their functional properties, as assessed by measurements of deformability and osmotic resistance, were not completely deteriorated.

Concentration-dependent effects of TBHP have been reported in previous studies. Specifically, it has been reported that erythrocytes incubated for 30 min with 0.5–1.0 mM TBHP exhibited only mild membrane impairment, whereas exposure to 3 mM TBHP resulted in more significant lipid peroxidative damage and alterations to membrane proteins [25]. Shorter incubation periods, such as 5 min with TBHP in concentrations of up to 3 mM, did not lead to significant hemolysis. However, a 2 mM TBHP concentration or higher was required to affect cation transport systems, likely by inhibiting sodium-potassium ATPase activity [22]. It is also possible to use lower concentrations of TBHP—e.g., lower than 1.5 mM. However, the incubation time in this case should be longer—4–5 h [29]. The prolongation of incubation time (1–6 h) could be potentially risky, as a positive correlation between the incubation time and hemolysis estimated via measurements of free hemoglobin levels was found [20].

The main part of this study is directed at evaluating the effects of melatonin across a range of concentrations on both control erythrocytes, as well as those that were subjected to oxidative damage induced by TBHP following melatonin incubation.

As mentioned in the Introduction, melatonin concentration in plasma varies significantly throughout the day. Circulating erythrocytes are exposed to melatonin in the bloodstream, and studies suggested a circadian rhythm in erythrocyte deformability in humans, with the lowest values observed in the morning [16]. Similarly, Yerer et al. [30] demonstrated the influence of different light/dark cycles on deformability in rats. Based on their findings, one may speculate that higher physiological plasma levels of melatonin may affect erythrocyte properties, potentially reducing their deformability. However, in vitro studies incubating erythrocytes with melatonin did not confirm this effect on deformability [16,17], which aligns with the findings of this study.

Contrasting results have emerged from experiments where oxidative stress was applied to erythrocytes alongside melatonin to investigate its effects. For instance, Dikmenoglu et al. [17] reported a significant deterioration in deformability when melatonin pre-treatment preceded oxidative stress induced by the combination of hydrogen peroxide and sodium azide. Conversely, Aydogan et al. [15] observed a protective effect of melatonin on erythrocyte deformability during oxidative stress induced by nitric oxide, whereas Yerer et al. [14] reported similar effects in the context of experimental sepsis. In addition, Morabito et al. [31] found that melatonin protects the band 3 protein in human erythrocytes against oxidative damage induced by hydrogen peroxide. Since the functional integrity of the band 3 protein influences erythrocyte deformability [32], its protection by melatonin may, in turn, promote better deformability. This study aligns with findings that report no effect of melatonin treatment on the deformability of intact erythrocytes while highlighting its ability to enhance deformability in erythrocytes exposed to oxidative damage after melatonin treatment. The antioxidant action of melatonin appeared to be less effective in erythrocytes already exposed to oxidative damage, as the melatonin concentration of 50 µM lowered DCF-fluorescence only in melatonin-preincubated erythrocytes that were then oxidatively damaged. Notably, in vitro experiments investigating the effects of various molecules, including melatonin, employ diverse experimental designs. In some studies, erythrocytes were pre-incubated with melatonin before being exposed to oxidative damage [17,31], while in others, the exposure to oxidative damage and melatonin occurs simultaneously [15,23,33,34]. In the present study, the beneficial effects of melatonin were less pronounced when administered after oxidative damage to erythrocytes. This finding aligns with a study that used hydrogen peroxide to induce oxidative damage in erythrocytes and quercetin as a protective agent. Pre-treatment with quercetin proved more effective than post-treatment, suggesting that not all oxidative stress-induced alterations are reversible, and the timing of antioxidant administration can significantly influence the results [19].

In addition to erythrocyte deformability, it should be emphasized that no effect of the incubation of erythrocytes with melatonin was observed on the ability of erythrocytes to survive in a hypotonic environment—i.e., erythrocyte osmotic resistance. Previously, it was shown that incubation of erythrocytes with 50 µM melatonin delayed the onset of 100% oxidative hemolysis induced by cumene hydroperoxide, extending the time from 180 min in the absence of melatonin to 330 min in its presence [23]. In another study, Krokosz et al. demonstrated a dose-dependent effect of melatonin within a concentration range of 0.02–3 mM on the inhibition of oxidative hemolysis induced by prolonged incubation in PBS. A significant protective effect of melatonin was observed at a concentration of 0.6 mM or higher [35]. These observations seem to be contradictory to the data produced by this study, but it could be a consequence of different hematocrit values that were adjusted in various experimental protocols. During in vitro experiments, especially when using oxidative agents, it is crucial to keep an exact hematocrit value. Hematocrit, preset during in vitro experiments, has a major impact on possible oxidative damage to erythrocytes. At a lower hematocrit value, the oxidant, e.g., TBHP, acts against lower antioxidant defense mechanisms. In contrast, a higher hematocrit value corresponds to a greater presence of antioxidant defense mechanisms that are characteristic of erythrocytes. Different experimental designs—such as in vitro experiments with varying values of hematocrit—could be also responsible for variations of observed effects. The hematocrit value preset in this study (i.e., 0.20) is relatively high in comparison with values around 0.01–0.02 in the aforementioned studies [23,35].

The measurement of oxidative stress in erythrocytes using the fluorescent probe DCF proved to be highly sensitive across all experimental protocols in this study. In addition to the dose-dependent effects of TBHP, the extent of oxidative damage was evident, as demonstrated by a consistent and statistically significant difference between the control and TBHP-incubated erythrocytes in both the pre- and post-stress melatonin incubation protocols. In both approaches—incubating erythrocytes with melatonin either before or after oxidative damage—the antioxidant activity of melatonin was observable when assessed with the DCF probe, although it was less evident in the case of melatonin incubation after oxidative damage.

Regarding the study’s limitations, monitoring additional oxidative stress markers in erythrocytes would provide a more comprehensive understanding of the complex effects of melatonin under various experimental conditions. Changes in erythrocyte deformability may also result from the modulation of ion channel activity [8], which could be at least partially influenced by melatonin [36].

It should be noted that the melatonin concentrations used in this experiment are supraphysiological. Plasma levels in the range of 25–1000 µM cannot be achieved through oral melatonin supplementation. However, the majority of in vitro studies utilize melatonin concentrations within this range [15,17,20,23,24,35,37,38]. Our findings are more applicable to in vitro settings, such as blood storage conditions. Previous studies have shown that melatonin is a promising agent in enhancing protective mechanisms against oxidative damage during storage [39,40]. The present study contributes to the knowledge required to optimize conditions for keeping the functional properties of erythrocytes.

Another significant finding of the present study is that melatonin intervention was ineffective in intact (i.e., control) erythrocytes. This observation aligns with previously reported findings indicating greater responsiveness of damaged erythrocytes to melatonin treatment, as demonstrated in patients with multiple sclerosis compared with healthy controls. Specifically, an increase in superoxide dismutase activity and a decrease in malonyldialdehyde levels in erythrocytes following oral melatonin supplementation were more pronounced in patients than in healthy volunteers [41].

## 4. Materials and Methods

### 4.1. Experimental Design

All procedures were conducted in compliance with the Declaration of Helsinki. The study was approved by the Ethics Committee of the Faculty of Medicine, Comenius University and University Hospital in Bratislava, Slovakia (date of approval: 2 February 2023, protocol code: 25/2023).

Venous blood samples were collected from healthy volunteers (n = 6, mean age: 24.44 ± 1.01 years) following an 8 h fasting period. Informed consent was obtained from all participants. Blood was collected using S-Monovette tubes (Sarstedt, Nümbrecht, Germany) containing ethylenediaminetetraacetic acid as an anticoagulant. The participants’ complete blood count was measured using the MINDRAY BC-6200 analyzer (Shenzhen, China), and selected parameters are presented in Table 1.

The blood was centrifuged at 1150 g for 5 min at 4 °C (Rotina 380, Hettich, Kirchlengern, Germany). Subsequently, the erythrocytes were washed three times with cold saline (0.9% NaCl). For all experiments, PBS containing glucose (in mM; NaCl 121.5, KCl 2.7, Na_2_HPO_4_ 10.1, KH_2_PO_4_ 1.8, glucose 5, pH 7.4, hereinafter referred to as PBS) was used for incubations and served as a solvent for preparing the TBHP and melatonin solutions. After each incubation, the erythrocytes were washed twice in PBS (360 g, 5 min). Erythrocytes were immediately diluted with PBS (*v*:*v*; 1:1) for further measurements. The overall experimental design is illustrated in Figure 1.

Firstly, the effect of different TBHP (458139, Sigma Aldrich, Saint-Louis, MO, USA) concentrations on erythrocyte properties was observed. Erythrocytes were diluted to a hematocrit of 0.20 and incubated at 37 °C for 30 min with constant shaking at 500 rpm, (BioSan Thermo-Shaker TS-100C, Riga, Latvia) using PBS with the following concentrations of TBHP (in mM): 0; 1; 1.5; 2; 2.5; 3; 3.5; and 4 (Figure 1a).

In the second part of the experiments, the effect of melatonin pre-incubation followed by oxidative damage was examined. A 1000 µM stock solution of melatonin (8.14537, Sigma Aldrich, Saint-Louis, MO, USA) was prepared daily, and working solutions of 25, 50, and 250 µM were made. The cells were incubated with melatonin for 30 min under the same conditions as in the first part. Subsequently, TBHP (final concentration: 2.5 mM) or an equivalent volume of PBS as a vehicle was added to each tube and incubated for another 30 min. For each melatonin concentration, a control and an oxidatively stressed aliquot were prepared. Afterward, the erythrocytes were washed twice in PBS and immediately processed (Figure 1b).

In the final part of the experiment, the effect of melatonin on erythrocytes already subjected to oxidative damage was investigated. Erythrocytes (hematocrit 0.20) were incubated with 2.5 mM TBHP or an equivalent volume of PBS for 30 min. Afterward, 1000 µM of stock melatonin solution (or PBS) was added to achieve a final melatonin concentration of 0, 50, and 250 µM in corresponding aliquots. The addition of melatonin reduced the final hematocrit of each sample to 0.15. After a 30 min incubation with melatonin, the erythrocytes were washed and prepared for measurement (Figure 1c) as previously described. In this part of the experiment, the lowest (25 µM) and highest (1000 µM) melatonin concentrations used in the second part were excluded. This decision was due to the time required for a series of incubations using each blood sample, which made it impractical to complete all experiments within an acceptable time frame. A complete set of melatonin pre-incubation and post-incubation measurements was conducted on the erythrocytes of each individual, without pooling the samples.

### 4.2. DCF-Fluorescence

Dichlorofluorescein (D6883, Sigma Aldrich, Saint-Louis, MO, USA) probe was used as a general indicator of oxidative stress. After incubation, 80 × 10^6^ erythrocytes from each sample were diluted in PBS at an approximate ratio of 1:9 (*v*:*v*). The cells were then exposed to DCF (final concentration 50 µM) for 30 min in the dark at 37 °C [42]. Fluorescence signals were measured using a Synergy H1 Hybrid Multi-mode Reader machine (Agilent, Santa Clara, CA, USA) with excitation and emission wavelengths set to 485 nm and 538 nm, respectively.

### 4.3. Erythrocyte Deformability

After each of the incubations was over, 20 × 10^6^ erythrocytes were used to determine erythrocyte deformability using a Laser-Optical Rotational Red Cell Analyzer (Lorrca MaxSis, RR Mechatronics, Zwaag, the Netherlands) as before [43]. This technique involves exposing erythrocytes to rotational forces between two concentric cylinders, within a medium of known viscosity, while applying controlled shear stress. Under these conditions, erythrocytes deform from their native biconcave disk shape to an elongated configuration. A laser beam is used to create a diffraction pattern, which is analyzed to calculate the Elongation Index (EI). The EI is determined by the formula: EI = (length − width)/(length + width), based on the dimensions of the elongated erythrocytes.

The results of erythrocyte deformability measurements are expressed using two parameters: EImax, the maximum elongation index, and SS1/2, the shear stress at which half-maximal elongation occurs. Additionally, the EImax-to-SS1/2 ratio was calculated and is presented in this study.

### 4.4. Erythrocyte Osmotic Resistance

Erythrocyte suspensions were mixed with NaCl solutions of varying concentrations (0.9, 0.7, 0.6, 0.55, 0.5, 0.45, 0.3, and 0%). First, an erythrocyte suspension with a hematocrit of 0.20 was prepared. From this suspension, 2 µL was transferred to a microplate, followed by the addition of 250 µL of the respective NaCl solution. After a 30 min incubation at room temperature, the microplate was centrifuged at 90 g for 5 min, and the supernatant was measured spectrophotometrically at 540 nm. The IC_50_ value, representing the NaCl concentration at which 50% hemolysis of erythrocytes occurs, was calculated from the data as previously described [42].

### 4.5. Statistical Analyses

Data are presented as means ± standard deviations. Data normality was tested using the Shapiro–Wilk test. The effect of different TBHP concentrations of erythrocyte properties was evaluated by one-way ANOVA with repeated measurements with Dunnett’s multiple comparisons test or mixed-effect analysis with Holm–Sidak’s multiple comparisons test (recommended by the GraphPad software to handle missing values).

For the evaluation of melatonin and TBHP effects, two-way ANOVA with repeated measures (or mixed-effect analysis) was used. Sidak’s (for ANOVA) or Tukey’s (for mixed-effect analysis) multiple comparisons tests (as recommended by GraphPad software) were used to assess the differences between groups. Significance was set at *p* < 0.05. All analyses were performed using GraphPad Prism 8.2.1 (GraphPad Software, San Diego, CA, USA).

## 5. Conclusions

This study supports the idea that the antioxidant action of melatonin can enhance erythrocyte functional status, as measured by erythrocyte deformability, but not osmotic resistance. However, this effect was observed only in cells subjected to oxidative damage after melatonin incubation.

## Figures and Tables

**Table 1:** Basic hematological parameters of volunteers.

Blood Parameter	Value	Blood Parameter	Value
RBC (×10^12^/L)	4.70 ± 0.50	WBC (×10^9^/L)	4.99 ± 0.85
HGB (g/L)	144 ± 15	Neutrophils (%)	50.0 ± 5.3
HCT (%)	42.9 ± 4.3	Lymphocytes (%)	40.2 ± 4.8
MCV (fL)	91.3 ± 1.2	Monocytes (%)	7.04 ± 1.75
MCH (pg)	30.7 ± 0.5	Eosinophils (%)	2.17 ± 0.74
MCHC (g/L)	336 ± 3	Basophils (%)	0.58 ± 0.11
RDW-SD	43.3 ± 1.9	Platelets (×10^9^/L)	229 ± 40

Data are presented as means ± standard deviations. Abbreviations: RBC—the count of erythrocytes, HGB—hemoglobin concentration, HCT—hematocrit, MCV—mean cell volume, MCH—mean cell hemoglobin, MCHC—mean cell hemoglobin concentration, RDW—red cell distribution width, WBC—white blood cells.

## Data Availability

The data supporting the findings of this study are available in this article.

## References

[1] Kim T., Slominski R.M., Pyza E., Kleszczynski K., Tuckey R.C., Reiter R.J., Holick M.F., Slominski A.T. (2024). Evolutionary Formation of Melatonin and Vitamin D in Early Life Forms: Insects Take Centre Stage. Biol. Rev..

[2] Reiter R.J., Tan D., Osuna C., Gitto E. (2000). Actions of Melatonin in the Reduction of Oxidative Stress: A Review. J. Biomed. Sci..

[3] Rodriguez C., Mayo J.C., Sainz R.M., Antolín I., Herrera F., Martín V., Reiter R.J. (2004). Regulation of Antioxidant Enzymes: A Significant Role for Melatonin. J. Pineal Res..

[4] Grivas T.B., Savvidou O.D. (2007). Melatonin the “Light of Night” in Human Biology and Adolescent Idiopathic Scoliosis. Scoliosis.

[5] Shirakawa S., Tsuchiya S., Tsutsumi Y., Kotorii T., Uchimura N., Sakamoto T., Yamada S. (1998). Time Course of Saliva and Serum Melatonin Levels after Ingestion of Melatonin. Psychiatry Clin. Neurosci..

[6] Domanski A.V., Lapshina E.A., Zavodnik I.B. (2005). Oxidative Processes Induced by Tert-Butyl Hydroperoxide in Human Red Blood Cells: Chemiluminescence Studies. Biochemistry.

[7] Cortese-Krott M.M. (2023). The Reactive Species Interactome in Red Blood Cells: Oxidants, Antioxidants, and Molecular Targets. Antioxidants.

[8] Radosinska J., Vrbjar N. (2021). Erythrocyte Deformability and Na,K-ATPase Activity in Various Pathophysiological Situations and Their Protection by Selected Nutritional Antioxidants in Humans. Int. J. Mol. Sci..

[9] Pernow J., Mahdi A., Yang J., Zhou Z. (2019). Red Blood Cell Dysfunction: A New Player in Cardiovascular Disease. Cardiovasc. Res..

[10] Zhou Z., Mahdi A., Tratsiakovich Y., Zahorán S., Kövamees O., Nordin F., Uribe Gonzalez A.E., Alvarsson M., Östenson C.-G., Andersson D.C. (2018). Erythrocytes From Patients With Type 2 Diabetes Induce Endothelial Dysfunction Via Arginase I. J. Am. Coll. Cardiol..

[11] Orrico F., Laurance S., Lopez A.C., Lefevre S.D., Thomson L., Möller M.N., Ostuni M.A. (2023). Oxidative Stress in Healthy and Pathological Red Blood Cells. Biomolecules.

[12] Chatzinikolaou P.N., Margaritelis N.V., Paschalis V., Theodorou A.A., Vrabas I.S., Kyparos A., D’Alessandro A., Nikolaidis M.G. (2024). Erythrocyte Metabolism. Acta Physiol..

[13] Podsiedlik M., Markowicz-Piasecka M., Sikora J. (2020). Erythrocytes as Model Cells for Biocompatibility Assessment, Cytotoxicity Screening of Xenobiotics and Drug Delivery. Chem.-Biol. Interact..

[14] Yerer M.B., Yapislar H., Aydogan S., Yalcin O., Baskurt O. (2004). Lipid Peroxidation and Deformability of Red Blood Cells in Experimental Sepsis in Rats: The Protective Effects of Melatonin. Clin. Hemorheol. Microcirc..

[15] Aydogan S., Yerer M.B., Yapislar H. (2004). In Vitro Effects of Melatonin on the Filtrability of Erythrocytes in SNP-Induced Oxidative Stress. Clin. Hemorheol. Microcirc..

[16] Vazan R., Plauterova K., Porubska G., Radosinska J. (2018). Changes in Erythrocyte Deformability during Day and Possible Role of Melatonin. Endocr. Regul..

[17] Dikmenoglu N., Ileri E., Seringec N., Ercil D. (2008). Melatonin Prevents Lipid Peroxidation in Human Erythrocytes but Augments Deterioration of Deformability after in Vitro Oxidative Stress. Clin. Hemorheol. Microcirc..

[18] Berker M., Dikmenoglu N., Bozkurt G., Ergönül Z., Ozgen T. (2004). Hemorheology, Melatonin and Pinealectomy. What’s the Relationship? An Experimental Study. Clin. Hemorheol. Microcirc..

[19] Remigante A., Spinelli S., Straface E., Gambardella L., Caruso D., Falliti G., Dossena S., Marino A., Morabito R. (2022). Antioxidant Activity of Quercetin in a H_2_O_2_-Induced Oxidative Stress Model in Red Blood Cells: Functional Role of Band 3 Protein. Int. J. Mol. Sci..

[20] Da Silva D.G.H., Chaves N.A., Miyamoto S., De Almeida E.A. (2019). Prolonged Erythrocyte Auto-Incubation as an Alternative Model for Oxidant Generation System. Toxicol. In Vitro.

[21] Skverchinskaya E.A., Tapinova O.D., Filatov N.A., Besedina N.A., Mindukshev I.V., Bukatin A.S. (2020). Investigation of Erythrocyte Transport through Microchannels After the Induction of Oxidative Stress with Tert-Butyl Peroxide. Tech. Phys..

[22] Dwight J.F.S.J., Hendry B.M. (1996). The Effects of Tert-Butyl Hydroperoxide on Human Erythrocyte Membrane Ion Transport and the Protective Actions of Antioxidants. Clin. Chim. Acta.

[23] Tesoriere L., D’Arpa D., Conti S., Giaccone V., Pintaudi A.M., Livrea M.A. (1999). Melatonin Protects Human Red Blood Cells from Oxidative Hemolysis: New Insights into the Radical-scavenging Activity. J. Pineal Res..

[24] Abdullah S.M., Rashid H. (2020). Melatonin Ameliorates BPA Induced Oxidative Stress in Human Red Blood Cells: An In Vitro Study. EMIDDT.

[25] Caprari P., Bozzi A., Malorni W., Bottini A., Iosi F., Santini M.T., Salvati A.M. (1995). Junctional Sites of Erythrocyte Skeletal Proteins Are Specific Targets of Tert-Butylhydroperoxide Oxidative Damage. Chem.-Biol. Interact..

[26] Trotta R.J., Sullivan S.G., Stern A. (1983). Lipid Peroxidation and Haemoglobin Degradation in Red Blood Cells Exposed to T-Butyl Hydroperoxide. The Relative Roles of Haem- and Glutathione-Dependent Decomposition of t-Butyl Hydroperoxide and Membrane Lipid Hydroperoxides in Lipid Peroxidation and Haemolysis. Biochem. J..

[27] Albertini M.C., Chibelli L., Ricciotti R., Fumelli C., Canestrari F., Galli F., Rovidati S., Bonanno E., Fumelli P. (1996). Morphological Alterations and Increased Resistance to Hemolysis in T-Butyl Hydroperoxide Incubated Rbc from Elderly Subjects. Arch. Gerontol. Geriatr..

[28] Baskurt O.K., Meiselman H.J. (2013). Data Reduction Methods for Ektacytometry in Clinical Hemorheology. Clin. Hemorheol. Microcirc..

[29] Besedina N.A., Skverchinskaya E.A., Shmakov S.V., Ivanov A.S., Mindukshev I.V., Bukatin A.S. (2022). Persistent Red Blood Cells Retain Their Ability to Move in Microcapillaries under High Levels of Oxidative Stress. Commun. Biol..

[30] Yerer M.B., Aydoğan S. (2006). The Importance of Circadan Rhythm Alterations in Erythrocyte Deformability. Clin. Hemorheol. Microcirc..

[31] Morabito R., Remigante A., Marino A. (2019). Melatonin Protects Band 3 Protein in Human Erythrocytes against H_2_O_2_-Induced Oxidative Stress. Molecules.

[32] De Almeida J.P.L., Freitas-Santos T., Saldanha C. (2012). Erythrocyte Deformability Dependence on Band 3 Protein in an In-Vitro Model of Hyperfibrinogenemia. Clin. Hemorheol. Microcirc..

[33] Zhao F., Liu Z.-Q., Wu D. (2008). Antioxidative Effect of Melatonin on DNA and Erythrocytes against Free-Radical-Induced Oxidation. Chem. Phys. Lipids.

[34] Zavodnik I.B., Domanski A.V., Lapshina E.A., Bryszewska M., Reiter R.J. (2006). Melatonin Directly Scavenges Free Radicals Generated in Red Blood Cells and a Cell-Free System: Chemiluminescence Measurements and Theoretical Calculations. Life Sci..

[35] Krokosz A., Grebowski J., Szweda-Lewandowska Z., Rodacka A., Puchala M. (2013). Can Melatonin Delay Oxidative Damage of Human Erythrocytes during Prolonged Incubation?. Adv. Med. Sci..

[36] Bhatti J.S., Sidhu I.P.S., Bhatti G.K. (2011). Ameliorative Action of Melatonin on Oxidative Damage Induced by Atrazine Toxicity in Rat Erythrocytes. Mol. Cell. Biochem..

[37] Tesoriere L., Allegra M., D’Arpa D., Butera D., Livrea M.A. (2001). Reaction of Melatonin with Hemoglobin-derived Oxoferryl Radicals and Inhibition of the Hydroperoxide-induced Hemoglobin Denaturation in Red Blood Cells. J. Pineal Res..

[38] Allegra M., Gentile C., Tesoriere L., Livrea M.A. (2002). Protective Effect of Melatonin against Cytotoxic Actions of Malondialdehyde: An in Vitro Study on Human Erythrocytes. J. Pineal Res..

[39] Şekeroğlu M.R., Huyut Z., Him A. (2012). The Susceptibility of Erythrocytes to Oxidation during Storage of Blood: Effects of Melatonin and Propofol. Clin. Biochem..

[40] Li S., Zhang L., Yuan H., Yang L., Song F., Liu H., Wei C., Ding H., Ma Q., Su Y. (2022). Effect of Low Concentration of Melatonin on the Quality of Stored Red Blood Cells in Vitro. Gematologiâ i Transfuziologiâ.

[41] Miller E., Walczak A., Majsterek I., Kędziora J. (2013). Melatonin Reduces Oxidative Stress in the Erythrocytes of Multiple Sclerosis Patients with Secondary Progressive Clinical Course. J. Neuroimmunol..

[42] Jasenovec T., Radosinska D., Jansakova K., Kopcikova M., Tomova A., Snurikova D., Vrbjar N., Radosinska J. (2023). Alterations in Antioxidant Status and Erythrocyte Properties in Children with Autism Spectrum Disorder. Antioxidants.

[43] Radosinska D., Jasenovec T., Golianova A., Szadvari I., Vazan R., Kovacicova I., Snurikova D., Vrbjar N., Radosinska J. (2024). Controlled Coffee Intake Enhances Erythrocyte Deformability, Na,K-ATPase Activity, and GSH/GSSG Ratio in Healthy Young Adults. Biomedicines.

